# Sound Matrix Shaping of Living Matter: From Macrosystems to Cell Microenvironment, Where Mitochondria Act as Energy Portals in Detecting and Processing Sound Vibrations

**DOI:** 10.3390/ijms25136841

**Published:** 2024-06-21

**Authors:** Daniela Valenti, Anna Atlante

**Affiliations:** Institute of Biomembranes, Bioenergetics and Molecular Biotechnologies (IBIOM), National Research Council (CNR), Via G. Amendola 122/O, 70126 Bari, Italy

**Keywords:** sound vibration, mitochondrial ATP production, sound sensitive mitochondria bioenergetics, cytoskeleton–mitochondria sound modulation, vibrational protein motions, elementary-particle sound

## Abstract

Vibration and sound are the shaping matrix of the entire universe. Everything in nature is shaped by energy vibrating and communicating through its own sound trail. Every cell within our body vibrates at defined frequencies, generating its peculiar “sound signature”. Mitochondria are dynamic, energy-transforming, biosynthetic, and signaling organelles that actively transduce biological information. Novel research has shown that the mitochondrial function of mammalian cells can be modulated by various energetic stimuli, including sound vibrations. Regarding acoustic vibrations, definite types of music have been reported to produce beneficial impacts on human health. In very recent studies, the effects of different sound stimuli and musical styles on cellular function and mitochondrial activity were evaluated and compared in human cells cultured in vitro, investigating the underlying responsible molecular mechanisms. This narrative review will take a multilevel trip from macro to intracellular microenvironment, discussing the intimate vibrational sound activities shaping living matter, delving deeper into the molecular mechanisms underlying the sound modulation of biological systems, and mainly focusing our discussion on novel evidence showing the competence of mitochondria in acting as energy portals capable of sensing and transducing the subtle informational biofields of sound vibration.

## 1. Introduction to the Therapeutic Power of Sound: Some Historical Notes

The language of the energy codes of living matter is made of sound, light, and vibration. Every living system in nature vibrates. We are immersed in an infinite oscillatory field and vibrate energetically at defined frequencies. From DNA to the brain centers, through receptors and cells, there is a continuous exchange of information through a huge vibrational flow. We are surrounded by vibrational sounds that mark the rhythms of our lives, and our body itself also continuously generates sounds. The heartbeat, for example, is a sound that accompanies and marks the entire course of our existence starting from fetal life, or the sound of respiratory rhythms.

Sound therapy represents an innovative approach that integrates multidisciplinary aspects of medicine, psychology, physics, and music [1]. This alternative therapeutic methodology is based on the notion of resonance. Normally, under the conditions of homeostasis, the electromagnetic fields surrounding the human body cells have their own “healthy” vibrational frequency.

The pioneering sound/cellular experiments, developed in 1981 by French musician/composer and acupuncturist Fabien Maman, photographically documented, for the first time under a microscope, the impacts of acoustic sound on human cells, showing the capability of human blood cells to respond to sound frequencies by changing their color and shape [2].

Since ancient times, sound has been used as a therapeutic tool. Greek physicians applied vibrations emanating from flutes, lyres, and zithers to improve digestion, alleviate mental disorders, and cure insomnia in their patients. In his ancient text De Anima, Aristotle (323–373 BC) referred to the beneficial effects generated by the sound of the flute, a practice also used for healing purposes by the ancient Egyptians. In their medical manuscripts on papyrus, the Egyptians mentioned the induction of “musical spells” through the use of a specific type of instrument that produced “ultrasound” to treat various pathologies. Pythagoras, the famous Greek philosopher and mathematician, also known as the Father of Harmony, systematically used sound as a therapeutic tool. He called this treatment “musical medicine”. Around 500 BC, Pythagoras and his students began using music to treat psychological problems, such as depression, aggressive behavior and anger.

In the yogic tradition, the use of “bijas”, the mystical “seed syllables” incorporated into chants and monosyllabic phrases known as mantras, is a well-recognized sound therapy. Mantras were used by Vedic thinkers in ancient India to calm the senses and mind. Specific mantras, chants, and sounds are used in Chinese qigong (or qi gong) to stimulate various organ systems in the body. An overview of the potential benefits of the “singing bowls” practice of sound meditation has recently been reviewed [3]. Singing bowl therapy has been defined as a holistic practice that utilizes the sound of special bowls made of metal alloys to promote relaxation, reduce stress, and support overall well-being.

The observation study by Goldsby et al. gave evidence of the beneficial effects of sound meditation, primarily singing bowl meditation, on stress, illness, and mental well-being in 62 women and men (average age, 50 years). The physical body, including nerve centers, circulatory systems, and cells that work with specific parts of the brain, responded well to the vibrations emitted [4].

The impact of music on the bioelectrical activity of the brain has been investigated in various contexts. As the famous British neurologist Oliver Sacks has asserted, “Our auditory systems, our nervous systems are tuned for music. Perhaps we are a musical species no less than a linguistic one” [5]. Human brain waves are oscillating bioelectrical activities of the brain that can be altered by various external stimuli. The effects of acoustic stimuli are among the most interesting and remarkable. Brainwave spectral analysis is a powerful tool for studies aimed at providing in-depth information on the effects of music on the brain. The human brain electroencephalogram (EEG) power spectrum, which is divided into at least five band waves associated with defined frequencies and corresponding to specific brain activities [6], is briefly summarized in Table 1.

Starting from a healthy population, several studies have also attempted to determine the effect of music in various pathological conditions such as disorders of consciousness, psychiatric diseases, and chronic diseases, as well as explore the role of music for rehabilitation purposes (for review, see [7]).

It is well known that music can change the humans’ emotional, physical, and mental state, and this phenomenon has been called the “Mozart effect”. But what is the Mozart effect? First described in 1991 by the French physician Alfred Tomatis in his book “*Pourquoi Mozart?*” [8], the Mozart effect is a phenomenon that signals an immediate and temporary improvement in individuals’ spatio-temporal abilities after listening to music. Tomatis used Mozart’s compositions in his attempts to remediate various disorders, such as learning disabilities, dyslexia, and attentional deficits, as he thought that the wide range of frequencies, intensities, and beats present in the music could improve performance in the auditory processing tasks [8].

**Table 1:** Human Brain Band Waves. The human brain EEG power spectrum is divided into at least five band waves that are classified, depending on their frequency expressed in hertz (Hz), as delta (1–4 Hz), theta (4–8 Hz), alpha (8–13 Hz), and beta (more than 13 Hz). Another category of very high (30–100 Hz) frequencies is referred to as gamma waves. Brain waves can be significantly altered when listening to sound. Thus, brain waves can change from a normal or even anxious state, like beta waves, to an exceptionally relaxed state (like alpha waves, theta waves, or even delta waves), while listening to relaxing music or through singing bowl sound meditation (for ref see [9]).

Band Waves	Frequency	Brain Activity	Wave Form
GAMMA	30–100 Hz	Gamma rhythmics are faster brain waves binding neurons and processing information from different brain areas.	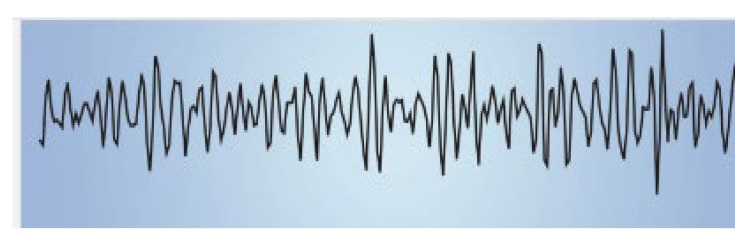
BETA	12–30 Hz	Beta waves are the predominant rhythmic activity of the frontal cortex and are associated with alertness, active concentration, but also anxious thinking.	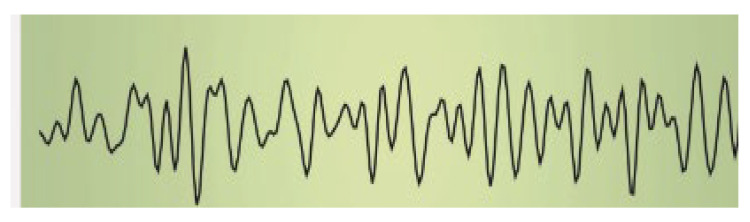
ALPHA	8–13 Hz	Alpha waves are the main rhythmics of the posterior regions of brain emerging during relaxation and resulting attenuated during brain stimulation.	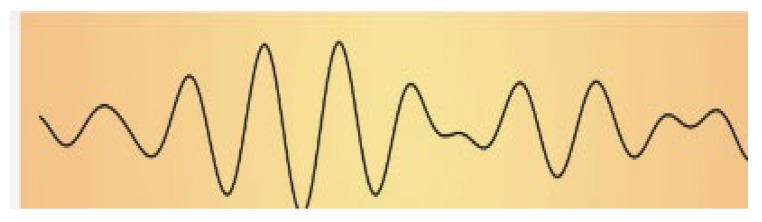
THETA	4–8 Hz	Theta waves are rhythmics associated with relaxed creative states and can be observed during meditation, light sleep, or deep relaxation.	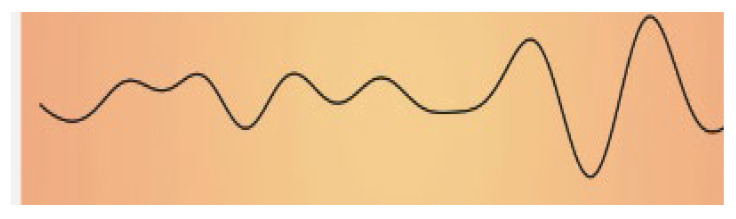
DELTA	1–4 Hz	Delta rhythmics are the highest amplitude and slowest waves, observed during deep dreamless sleep, or deep states of meditation.	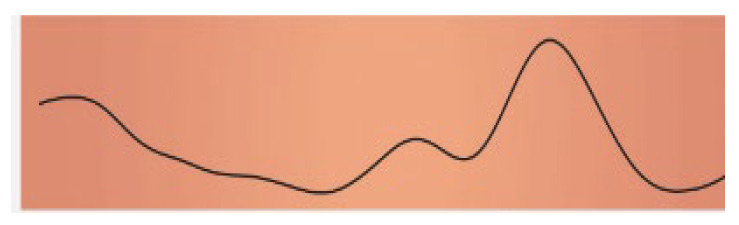

Shortly after the publication of Tomatis’ book, in 1993, physicists Frances Rauscher and Gordon Shaw published a study in Nature entitled “Music and spatial task performance”, according to which listening to the Sonata in D Major for two pianos (KV 448) by Wolfgang Amadeus Mozart produced a temporary increase in spatial reasoning abilities in the volunteers [10].

**Figure 1:**
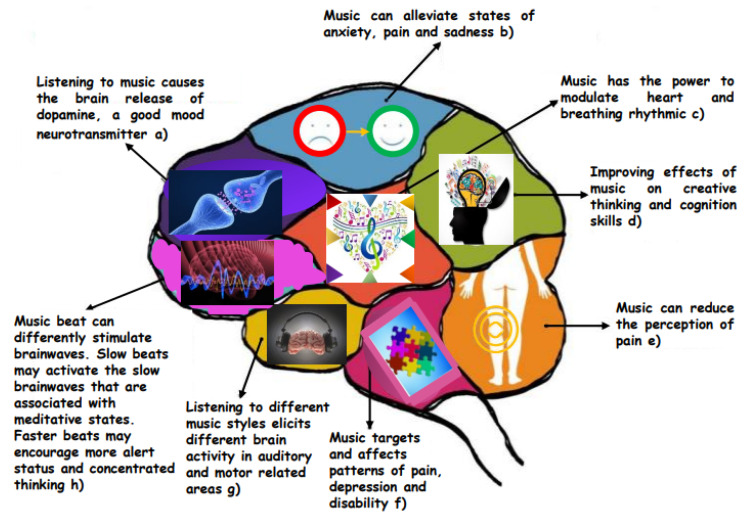
Brain on Music: How Sound Can Impact Brain Activities (a) Salimpoor et al., 2011 [13]; (b) Nilsson, 2008 [14]; (c) Kulinski et al., 2022 [15]; (d) Eskine et al., 2018 [16]; (e) Tan et al., 2010 [17]; (f) Siedliecki et al., 2006 [18]; (g) Martín-Fernández et al., 2021 [19]; (h) Mofredj et al., 2016 [20].

Scientific studies investigating the effect of music on the brains of healthy people have often used the Sonata K448 by Mozart. Several years later, in 2001, John Jenkins, professor at the University of London, returned to the topic and, in the Royal Society of Medicine, published the article “The Mozart effect”, containing a series of studies to verify the therapeutic effects of music. By having patients suffering from epilepsy listen to Mozart’s Sonata K448 for ten minutes a day, he noticed that this exposure had the effect of a drastic reduction in epileptic attacks [11]. Other studies had confirmed that epileptic children who had been given piano lessons for six months had higher scores on movement tests than other children who had been taught to use the computer [12]. Why Mozart? According to current literature, this sonata causes a significant increase in relative alpha band power, as well as in the median frequency of background alpha rhythm in young adults and healthy elderly people. There are different theories of thought on these effects; for some researchers, it depends on the shades used, while for other scholars, it is due to the periodicity of Mozart’s structure. “The Mozart effect” is therefore a courtly musical language capable of increasing the elasticity of the neural circuits of the cerebral cortex, strengthening the creative processing of the right hemisphere, associated with space–time reasoning. The scientific orientation leads us to believe that this effect is triggered by a certain musical language and is not comparable to what alphabetic writing can provide. Unlike verbal language, there are many areas of the brain activated by musical stimuli (Figure 1). In fact, music produces auditory stimuli articulated in a very complex way. The brain processes sound and music in a hierarchical and distributed way, therefore activating perceptual processes that occur simultaneously in different brain areas, even very distant from each other.

Towards the end of the nineteenth century, researchers began to conduct systematic studies of sound or music in medicine and clinics. In recent years, music therapy has been widely used in nursing homes, hospitals, and clinics where it is now used to treat dementia [21], cancer [22] and pain [23], as well as for palliative care [24].

In this narrative review, we will undertake a multilevel path from the macrocosm to the cellular and molecular microenvironment, discussing the intimate vibrational sound activities that permeate and shape living matter, and the power of sound on biological systems, mainly focusing our discussion on recent research evidence showing the competence of mitochondria to act as energetic portals capable of sensing and transducing the subtle informational biofields of sound vibration.

## 2. The Sound Vibration at the Basis of Universe Structure: From Stars to Cells

Vibration and sound are primordial structures at the basis of the universe. From a physical point of view, sound is essentially the propagation of a wave, which, in turn, is a disturbance of the system that transfers energy through space. Therefore, it is reasonable to assert that sound is an energy transport system; in fact, it propagates in the form of pressure waves in the air. When this energy encounters a medium other than air, it propagates differently, inducing oscillatory motions in the system and becomes vibration. Sound is essentially vibration. The word “vibration” comes from the Latin “vibrationem”, which means shaking. When an object is disturbed by a mechanical stimulus and begins to oscillate and vibrate, sound energy is produced [25]. This energy propagates in the form of pressure waves coming from the sound source, usually as a longitudinal wave consisting of compressions and rarefactions of matter [26]. Sound waves can travel through different media, including gases, liquids, and solids, with speeds that depend on the medium [27]. Typically, sound travels faster in a liquid or solid medium (e.g., water or a steel bar) than in a gaseous medium [26,28].

According to the intrinsic vibrations of the medium with which the wave comes into contact, we may have constructive or destructive interference phenomena, conditions in which there is, respectively, an amplification or reduction in the vibration and therefore of the transmitted energy. When there is constructive interference and therefore an increase in vibration, it is said that there is resonance. A resonance phenomenon typically causes a significant increase in the amplitude of the oscillations, which corresponds to a notable accumulation of energy within the stressed system.

It is important to specify that it is only the body with a frequency similar to the resonant one that increases its vibration, not bodies with different vibrations. For example, the human body does not vibrate as a single mass, but each individual part has its own resonance frequency (for review, see [29]).

**Figure 2:**
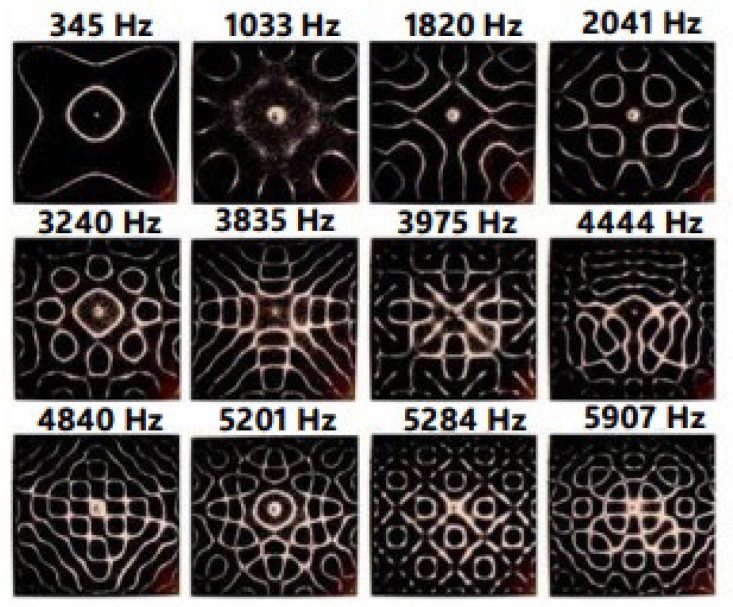
Cymatics: Study of Wave Phenomena and Vibration. All sounds we hear are waves, typically in the air, that vibrate your eardrums, which, in turn, create electrical signals that are sent to your brain for interpretation. These waves are only invisible to our eyes simply because the air is invisible. The concept of cymatics is to use a substance other than air to transmit sound to visualize the waves. This was most notably performed by Hans Jenny, who attached a sine wave frequency generator to a metal plate, placed lycopodium powder on the metal plate, and then vibrated the metal plate at different frequencies using the frequency generator. He found that at certain frequencies, the sand or dust would form geometric patterns that became more and more complex at higher and higher frequencies [30].

The experiments of the scientific discipline called cymatics clearly highlight the effect of sounds and vibrations on matter [30]. Cymatics demonstrates that sound waves have an intrinsic order in which they manifest themselves and when crossing matter, they arrange it in ordered shapes and geometric structures of considerable complexity (Figure 2). It is therefore important to consider that this order is also reproduced within living organisms. It is now a common notion even for modern science that the order and their intrinsic structural complexity are determining elements of living organisms.

Sound is the shaping matrix of the entire universe, so it is intuitive to understand how reality is permeated by vibrations and sounds, and modern physics not only scientifically confirms this intuition but also says that reality is intrinsically made up of vibrations.

According to String Theory, for example, it is the vibrational state of a string that determines the properties of matter. String Theory assumes that the fundamental forces of nature can be considered as vibrating one-dimensional strings. They have an infinitely small size, at the level of Planck’s Constant (10–35 m), they propagate in space and interact with each other constituting the network of reality. In fact, at a dimensional level higher than Planck’s Constant they appear as normal particles, with mass, charge, and other properties which are, however, determined by the vibrational state of the String [31].

The microscopic network of the universe appears to be an intricate multi-dimensional labyrinth in which strings vibrate continuously, giving rhythm to natural laws, and in which the fundamental properties of objects are not arbitrary, but are derived from this complex structure that orchestrates the evolution of the cosmos. Similar to the vibrations of the strings of a guitar or violin, which generate sounds accompanied by all the higher harmonics, so do the strings give rise to the different sounds and harmonics which manifest themselves both in nature and in our measuring instruments in the form of protons, neutrons, electrons up to the infinitesimal part of the perceptible.

The frequencies radiated by atoms are a globally interrelated polyphony (electroweak, magnetic, and gravitational energy) which produces different sound frequencies and allows information to express itself.

According to String Theory, matter is vibration [31]. This vision and the therapeutic power of sound vibration has already been present and incredibly clear in antiquity [32]. In fact, in every system of traditional knowledge, sound, word, and vibration are considered the roots of creation and the sustenance of the universe. According to the Vedic conception, the Universe is made up of five elements or states of matter which have been derived from the primordial imbalance in the form of the sound OM. Sound is the primordial energy that organizes itself into the forms of material reality.

We tend to have a very “human-centric” perspective of sound, as we otherwise define sounds that are above and below our hearing range.

Sound represents a mechanical force with the frequency property expressed in Hertz (Hz), defined as the number of times a wave or vibration repeats in one second.

The distance between each repeating wave, known as the wavelength, decreases as the frequency increases. The relationship between frequency and wavelength remains the same when sound travels at the same speed.

This gives rise to the classical formula relating the speed of sound, *c*, to the frequency, *f*, and wavelength, *λ*, as follows:

*c* = *fλ*

Sound travels through different materials at different speeds, as the structure of each material can affect its propagation. Generally, denser and more regular structures transmit sound more quickly, so sound usually travels faster in liquids than in gases. Due to the change in speed, the waves will move away. Therefore, when a wave of a specific frequency travels in a material of a different density, the speed and wavelength will change. Everything in nature vibrates at different frequencies. Quantum physics describes the universe as nothing more than vibrating strings of energy! Scientific research has shown that different parts of our body have their own sound signature [29]. When parts of the body are stressed or diseased, they no longer produce the correct sound wave.

**Figure 3:**
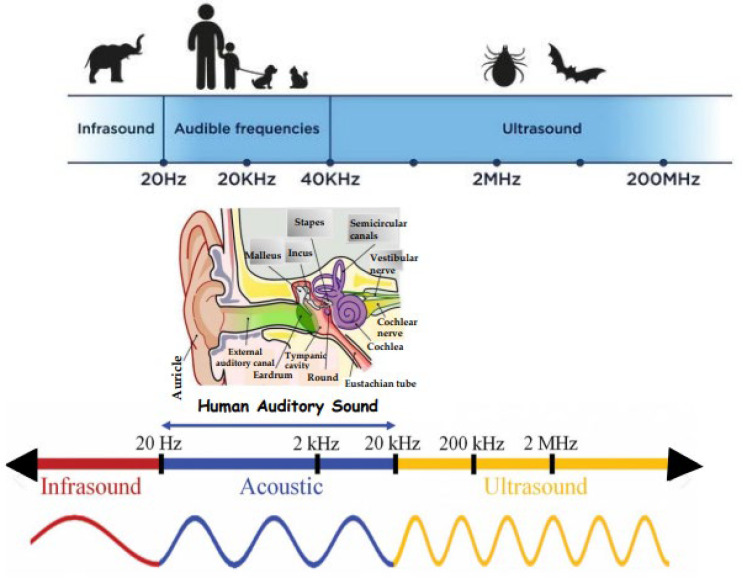
An Overview of the Entire Spectrum of Sound Frequencies and Wavelengths.

The entire spectrum of sound waves covers a very wide range of frequencies, of which human audible frequencies occupy a very small portion from 20 Hz to 20 kHz (Figure 3). At one end of the scale, we can detect sound waves at very low frequencies around black holes and stars that provide information on their dynamics [33]. At the other end of the scale, we can measure the nuclear vibration of molecules at terahertz (THz) frequencies [34].

## 3. The Modulating Power of Sound Vibration on Biological Systems

The effects and therapeutic action of vibrations on the human body applied through sounds, beatings of objects on the body, visual effects, and colors have been known since ancient times. Modern medicine has only recently begun to use sound and vibrations in the therapeutic field [35,36,37] and, above all, for rehabilitation purposes [38] or for diagnostic purposes [39]. In recent times, low-frequency vibrations have found therapeutic applications in the field of Vibroacoustic Therapy (VAT) with interesting results in various pathological conditions [40].

However, numerous pieces of experimental evidence have highlighted the different effects induced by sound and vibrations on biological organisms as a whole and tested the diffusive power of sound at the cellular level (for a systematic review, see [41]), even though, according to conventional medicine, they have no proven therapeutic effects.

Cells vibrate and communicate incessantly with each other through a sound language that science has been beginning to codify in recent years, sensing new therapeutic potential. Each part of our body has its own resonance frequency which corresponds to the state of health, and when the frequency is detuned, the disease manifests itself [29].

Sound is in us, it constitutes us, builds us, and constantly passes through us, transporting information inside and outside of us through that wonderful element that is water, which makes up over 70% of us and in which sound propagates faster than in the air.

Interestingly, prenatal sound is emerging as an alternative source of information for embryos, in addition to the well-known effects of maternal or environmental biochemical signals. From invertebrates to birds, embryos use external acoustic cues to adaptively modify their developmental courses [42]. Prenatal sound and vibrations can shift hatching time in all oviparous taxa, but also directly impact individual cognition and physiology [42]. This may occur through neurological or epigenetic changes [42], or even through direct alteration of cell physiology in plants [43,44]. The use of sound is not, in fact, just an animal prerogative; it is used by all living beings to communicate, adapt and evolve. Recent research has, in fact, shown that plants use sound to communicate with each other and to explore the environment. Gagliano et al. demonstrated that plants are able to use sound waves to detect the presence of water at a distance and then direct their roots in the direction of the water [45].

### 3.1. The Diffusive Power of Sound at the Cellular Level

Music, through the diffusive power of sound, deeply permeates our body. Our cells, in turn, produce their own identifying sound signatures which can give information on their state of health or disease.

**Figure 4:**
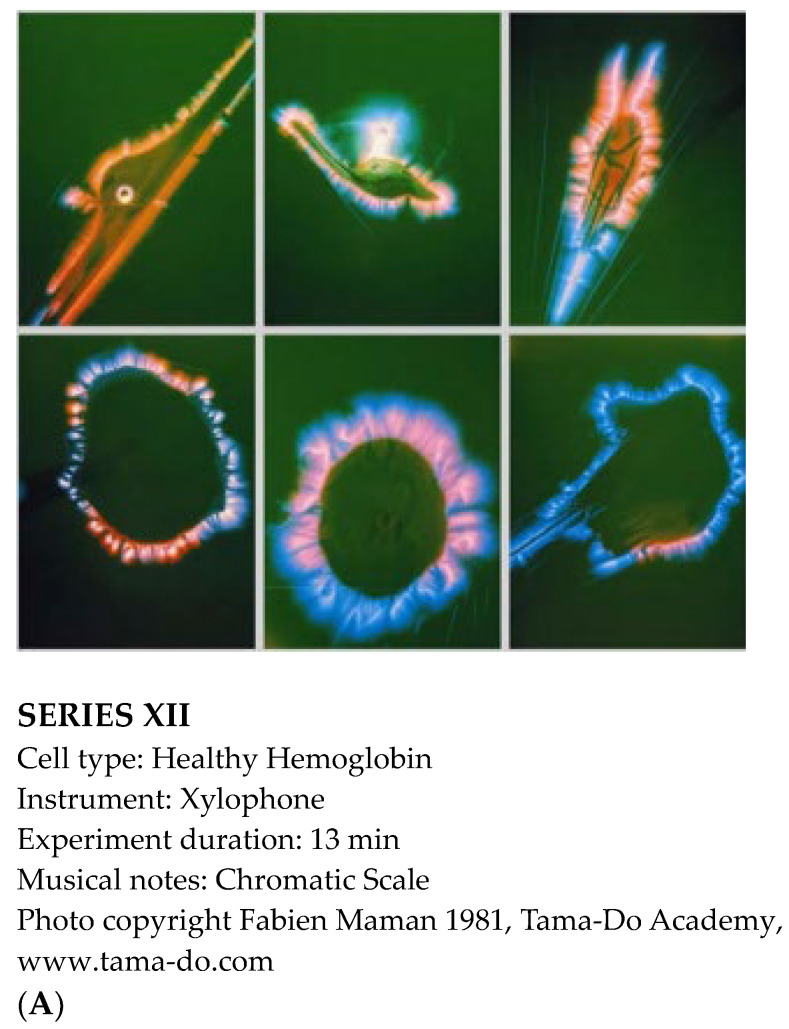

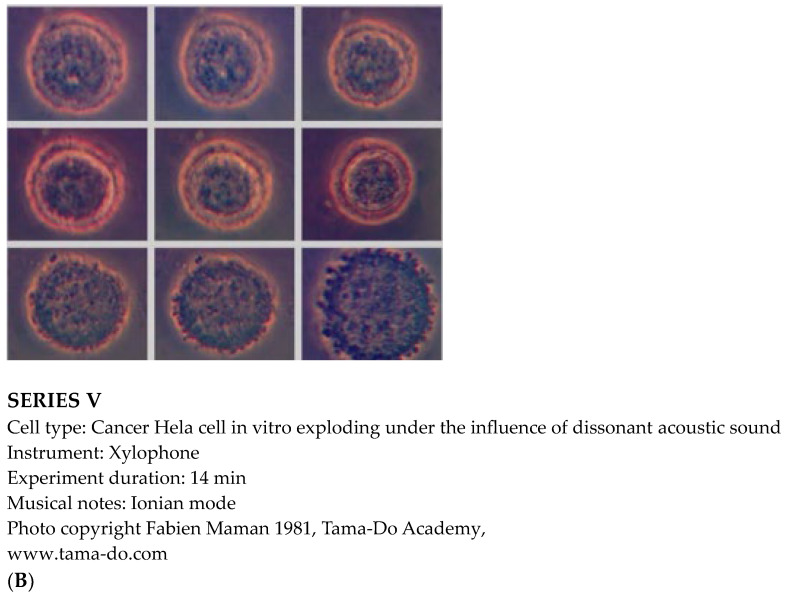
The Power of Sound at the Cellular Level. The photos shown are reproductions of those from Maman’s book [46] with permission from the Tama-Do Academy, www.tama-do.com. (**A**) For this series of photographs, healthy human blood cells were used while playing the chromatic scale with a xylophone, in order to observe the change in the field around the cells. One photograph was taken per note per minute. For each note, there appeared a specific shape and color directly related to the frequency of the sound. (**B**) For this series of experiments in human Hela cancer cells, the Ionian scale on the xylophone was used by playing in sequence the following notes: do–re–mi–fa–sol–la–ti, and then do–re at the next octave. One photograph was taken per note per minute. Fourteen minutes was enough time to destroy the Hela cancer cell when these nine different frequencies were used.

“In the heart of the cells, in the spiral of DNA, is written the divine history. When scientific research, spiritual practice, and artistic expression work together, heaven and earth are in resonance. This is the vibratory promise that is the gift of our musical universe”, wrote Fabien Maman, one of the world’s leading experts of vibrational sound. In the early 1980s, he conducted pioneering sound–cellular biology experiments at the University of Jussieu in Paris, showing the impacts of acoustic sound on human cells and their energy fields. Maman’s research photographically documented, for the first time under a microscope, that acoustic sound can revitalize human healthy cells and destroy cancer cells [46] (Figure 4). He also found that healthy cells changed shape and color according to the pitch and timbre of each note, and that when cells felt a ‘vibratory affinity’ with a certain note, the cells’ aura transformed into a mandala shape of vibrant color, such as pink and blue (Figure 4A).

While working with human cells, Maman found that Hela cancer cells, when exposed to a series of dissonant notes, exploded rather than vibrated (Figure 4B).

During the same period as Maman’s research, he met French physicist Joel Sternheimer, who had discovered the vibratory frequency of elementary particles. Long before cosmologists developed the “String Theory”, Sternheimer was transposing certain molecular structures into musical patterns, creating “the music of the molecules” [47].

In his brevet accepted by the National Center for Scientific Research in Paris, Joel Sternheimer provided evidence that to each atomic particle, there corresponds a frequency which is inversely proportional to its mass. This “music” of the elementary particles means that we, who are composed of these elementary particles, are also composed of musical frequencies. Sternheimer found that if there was a fault in an organic structure, the molecules of that structure did not vibrate. Maman’s sound cellular research came to the same conclusion. Both Sternheimer and Maman concluded that whereas sick cells lacked flexibility and resonance, healthy cells vibrated when they recognized their fundamental resonance [46,47].

Sound frequencies can deeply influence the behavior of different cell types and subsequently the functions of different organs [48,49]. The pressure waves produced by sounds can affect cells or their structures by determining micro-vibrations, or even generate resonances, i.e., the synchronization of biomolecular oscillatory patterns within cells [50]. Furthermore, acoustic vibrations in the form of a single frequency [51], noise, or music have been shown to influence proliferation, viability, and hormone binding in human cell cultures [52,53] and animal models [54,55]. Acoustic stimuli are therefore of fundamental importance in regulating the spatial interaction between cells, modulating both their individual life and collective behaviors [56,57], and their intracellular and intercellular organization, which are crucial elements that control their function [58]. Studies by Lestard et al. planned to better value the direct effects of acoustic vibrations, in the form of music, in cultured human cells. Their results suggest that the mechanisms of cell growth arrest and/or cell death induced by acoustic vibrations are similar for auditory and nonauditory cells [52,53].

Ventura and colleagues demonstrated and patented, for the first time, the ability of cells to express the “vibrational signatures” of their state of health and their differentiation potential [59]. Using an atomic force microscope (AFM), which is capable of measuring the structures and properties of living cells at the atomic level, they discovered that each living cell produces a pattern of vibrations that changes depending on the task performed by the cell. “Sonocytology” is the term introduced to identify a new area of research based on evidence that, after a careful amplification process, cellular vibrations recorded with AFM can be transformed into audible sounds, providing an accurate assessment of the functional properties of the cell [59]. More recently, Ventura et al. provided evidence that human stem cells—residing in all body tissues and managing tissue repair—are skilled at responding with various vibrational signatures to the sounds produced in the form of music or voices in live performances [60]. Human stem cells reacted to the diverse frequency patterns of the performer’s voice with different spectral emissions. The possible biological implications of these results have yet to be established. It is well ascertained that a wide variety of biological processes are influenced by the nanomechanical properties of subcellular structures. An example of this is that the vibrational modes generated by the cytoskeleton and nucleo-skeleton, whose resonance patterning imparts features characteristic of connectedness, can be transmitted up to and recorded from the cell surface [61]. Several studies have suggested that the response to sound stimulation is complex, further confirming that cell types other than auditory cells can react directly to sound stimuli. For example, it has been shown that sound wave stimulation brings about significant changes to the protein structure of tobacco cells, producing an increase in the alpha helix structure and a decrease in the unwound β helix [62]; furthermore, sound stimulation has produced effects on the cell cycle and on the growth of plants [63,64]. It has been demonstrated that bacterial cells are able to respond to specific single acoustic frequencies and are capable of emitting sounds [65]. Sounds of 1 kHz and 5 kHz have been shown to promote the growth of *Escherichia coli* [66,67].

### 3.2. The Power of Sound at the Subcellular Level: Effects of Music on Mitochondrial Bioenergetic Function

Now, let us continue our travel into the power of sound by going down to the subcellular level, focusing on the effects of sound on the multifunctional cellular organelles identified as mitochondria. Traditionally recognized as the cell’s powerhouse, mitochondria are dynamic, energy-transforming, biosynthetic and signaling organelles that actively transduce biological information [68]. A new dynamic framework is emerging that considers mitochondria as cell processors thus defining their signal transduction activity as the mitochondrial information processing system (MIPS) [68]. Recent studies have investigated whether and how the mitochondrial function of mammalian cells can be modulated by acoustic vibration, given the competence of mitochondria to act as a gateway to receive, process, and integrate energetic stimuli and information fields [69,70,71].

The effects of different sounds and musical styles on cellular function and mitochondrial activity were evaluated and compared in several types of cultured mammalian cells, investigating the underlying mechanisms responsible [72,73,74].

In their pilot study, Del Lin et al. exposed cell cultures of murine atrial cardiomyocytes (HL1) to 20 min sound sequences. As examples of sounds, such as meditative music, melodies with high frequencies, high rhythm sounds, combined urban traffic noises, and human screams, were used [72,73], certain cellular behaviors, such as contractions, vesicular traffic, indirect movements of the cytoskeleton, and mitochondrial activity, directly in response to each different acoustic stimulus were followed, acquiring a time-lapse video with frames taken every 100 ms. Subsequently, changes in the cytoskeleton were evaluated by immunofluorescence analysis. Finally, to explain the results, the geometric characteristics of the waveforms of the sounds used were photographed and filmed. The results of this qualitative analysis appear to draw attention to a different pattern of cellular growth as a function of definite sound stimuli, with a different expression pattern and organization of cytoskeletal proteins and mitochondrial activity [72,73]. Different sound stimuli have been shown to affect the contractility and the spatial organization of HL1 cells, resulting in a different localization and fluorescence emission of cytoskeletal proteins. The cellular behavior of HL-1 cells also appears to change based on the sound stimulus that impacts their surface, where certain acoustic stimuli are able to enhance contractility by increasing vesicular traffic within them, while others compromise it, leading to cell lysis. The authors assume that the meaning of the sound vibrations applied can be encoded in their specific geometric shape, and that the sound wave trains characteristic of each acoustic vibration could directly modulate the morphology of the cytoskeleton [75] by mechanical stress or sending relaxation signals up to the nucleus, thus driving and positively or negatively influencing the trafficking and bioenergetics of the mitochondria [76], as well as the turnover of cellular microvesicles [77], which normally control the oscillatory movements of proteins present in the cytoplasm [78]. The mechanical pressure forces exerted by vibrational sound waves on cellular adhesion receptors such as integrins and cadherins can be rapidly channeled along cytoskeletal filaments and directed to distant sites in the cytoplasm and nucleus [79,80,81], altering the activities of the cellular genome [82]. Microtubules represent an important protein component of the cytoskeleton and are responsible for the structure and shape of the cell and its movements. Microtubular proteins can also interact with other cytoplasmic organelles, such as the mitochondria, regulating their functions [83,84]. It is believable that there could be an intimate relationship between acoustic wave trains and proteins [85], given that proteins in the human body vibrate in different patterns, like long-range vibrational modes [86], and can also interact through quantum interference modalities [87].

A recent study by Feng et al. offers experimental evidence in favor of the hypothesis that music could resonate directly with human cells, modulating their mitochondrial functions differently [74]. In their analysis, the effects of Chinese five-element music with two types of Western music (heavy metal and classical) were compared based on the mitochondrial bioenergetic function, oxidative damage, and cellular growth of in vitro cultured HEK293T (human embryonic kidney) cells [74]. The five-element music, also called five-tone music, originates in traditional Chinese medicine, which has been shown to play an important role in the prevention and treatment of several diseases [88]. The five tones refer to “Gong, Shang, Jiao, Zhi and Yu”, which correspond to five organs of the human body, namely the spleen, lung, liver, heart and kidneys. Just as in traditional Chinese herbal medicine, the five tones are produced by combining different instruments, rhythms, forces, and harmonies [88]. The results of Feng et al. showed that the effects of such music could be positive or negative with respect to the cell growth and mitochondrial bioenergetics of the HEK293T cells, in correlation to the different musical styles [74]. In particular, the classical music and five-element music were shown to stimulate cell growth and the antioxidant capacity of cells, while five-element music has exclusively been found to significantly increase mitochondrial energy efficiency by hugely increasing mitochondrial ATP synthesis and drastically reducing the production of reactive oxygen species (ROS). Five-element music significantly increased ATP levels by 17%, while no significant differences were found between the other two musical styles. Conversely, heavy metal music negatively affected cell viability and increased ROS production [74]. Regarding antioxidant capacity, a significant reduction in the activity of the antioxidant enzyme superoxide dismutase (SOD) was measured after treatment of the HEK293T cells with heavy metal music, while no significant change in SOD activity was detected in the cells exposed to both the classical and five-element music [74]. Conversely, a significant increase in the GSH levels was observed in the cells exposed to treatment with both the classical music and five-element music (8% and 21%, respectively), while no significant change in GSH levels was produced by cell treatment with heavy metal music. The observed increase in GSH levels in response to five-element music is congruent with the observed reduction in ROS levels. These results indicated that different styles of music have unique effects on antioxidant capacity.

This study investigated the mechanisms involved in the interaction of music at a cellular/subcellular level without the interference of complex human perceptions and personal musical tastes. Music contains specific vibrational frequencies and harmonic structures. The different musical styles include their own identifying features of rhythm, melody, strength, speed, and specific vibrational frequencies and harmonic structures. The spectral analyses of sound frequencies in all three types of music were compared and the potential mechanisms involved were explored. Interestingly, although both five-element music and classical music showed quite similar spectral distribution of sound frequencies, their revealed biological effects were comparable only with respect to cell growth [74]. Therefore, the results seem to indicate that five-element music has a more selective improving effect on human cell physiology and mitochondrial bioenergetic function than classical music. From a physical point of view, the waveforms and frequencies of music that were measured, and the uniqueness of the frequency sound signatures for each type of music, could be contributing factors to the different biological effects observed on cell growth and mitochondrial function. Five-element music, specifically, has been observed to contain ultralow frequencies (<50 Hz) that are absent in the other types of music tested that could be responsible for its selective improving effect on mitochondrial function [74].

The possible mechanism involved in the musical-style-dependent selective increase in the ATP pool measured exclusively in cells that were exposed to five-element music has not been investigated and is currently unknown. Some of the following hypotheses can be put forward: Mitochondria could act as dynamic bio-informational gateways that are able to sense and selectively process the different frequency sound signatures by accelerating the electron transfer of the mitochondrial respiratory chain due to changes in the redox properties of the electron carriers following the sound modulation of their electronic states, thereby causing greater efficiency of ATP generation by the process of oxidative phosphorylation. Mitochondrial processing of sound frequencies could also be transduced and transferred outside the mitochondria and across the cell at the nuclear level, modulating the expression of mitochondrial respiratory complexes differently.

In very recent studies, Feng et al. extended their exploratory investigations, showing, for the first time, the effect of information fields also generated by written Chinese words or texts on mammalian kidney cells in tissue culture [89,90]. Feng et al. reported that there were improving biochemical effects generated from multiple individual Chinese words with positive meanings on cultured human kidney cells [90]. The effects were further evaluated in cells exposed to stress due to exogenous hydrogen peroxide supplementation and during repair following oxidative damage [90]. When the cells were treated with hydrogen peroxide for 3 h, whole-cell ATP levels in the positive text group were increased by 21%, accompanied with a 29% reduction in ROS when compared with the control group. Furthermore, the positive texts significantly increased the ATP levels of the repaired cells (24 h after peroxide-induced cell damage) by 17%, and decreased ROS levels by 15%. Therefore, the information field of words with a positive meaning appeared to protect the cells from oxidative damage and produced faster repair. In some cases, this effect has been associated with a decrease in ROS and an increase in cell growth, indicating an improvement in the antioxidant capacity of the cells. The authors proposed that mitochondria may act as antennas to sense the spatial information field generated by geometric patterns and word texts [89,90]. Further research will be needed to understand resonance and the biological mechanisms involved in cellular signal transmission and reception. This could open a new research direction to explore the potential health benefits of information fields associated with words.

### 3.3. From the Cell Microenvironment to Molecules: Sound of Protein Vibration

Finally, we conclude our discussion on the multidimensional sound vibrational activity of living systems by moving to a molecular level and briefly mentioning the vibrational properties of proteins. Protein vibrations can cover a wide energy range, from the far infrared (IR) over the THz range (30 cm^−1^) to the near IR, typical for the stretching vibrations of covalently bonded hydrogen atoms [91]. It has long been speculated that protein structural change, which is critical to their function, is mediated by long-range vibrational motions involving dynamic networks that extend throughout the protein [92,93]. Each amino acid constituting protein has its own vibratory frequency. These frequencies can be transformed into sounds thanks to sonification, a process that translates a set of inaudible vibrations into audible sounds. Markus J. Buehler and his team at the Massachusetts Institute of Technology developed this sonification method to associate each of the twenty amino acids with an audible frequency. Starting from the observation that amino acid vibrations range in a frequency spectrum of between 0 and 25,000 Hz, they developed an algorithm to make the frequencies audible and last a few milliseconds. The results obtained faithfully reflect the biochemical characteristics of the corresponding proteins [94].

## 4. Some Concluding Remarks

The language of the energy codes of living matter is made of sound, light, and vibration. In this narrative review, a brief excursus has been made into the complex sound, vibratory, and resonant network that appears to permeate and constitute the most intimate dimension and common thread that binds and interconnects the physical universe and the living matter, extending from the macrocosm to the cellular and molecular microcosm.

Growing experimental evidence highlights the existence of a vibrational bioinformation regulation system working across subcellular and cellular levels to entire living systems [95].

**Figure 5:**
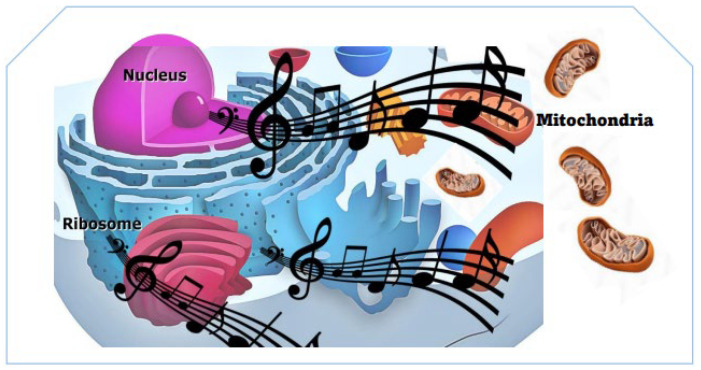
The Diffusive Power of Musical Sounds at the Cellular Level.

The sound of music is a vital and basis component of living systems, representing the core of the human experience and an important part of human evolution, deeply rooted in our biology [96]. Music, through the diffusive power of sound, deeply permeates the cells in our body (Figure 5).

The life rhythm of biological systems works as a symphony of oscillatory patterns, where sound vibration can modulate gene expression for biological signaling [95]. A new dynamic framework is developing within biological systems, which appear to be modeled on the vibrational networks and patterns capable of orchestrating and driving protein expression in specific trajectories, through mechanical/electromagnetic oscillatory rhythms of the cytoskeleton and mitochondria, subsequently moving towards the molecular level to follow the vibratory motions of proteins [95]. These vibrational phenomena modulate and influence crucial traits of cellular life. In addition to the established capability of cells to detect and signal each other’s presence even in the absence of physical contact [97], suggesting some rudimentary form of cellular “vision” [98], cells can also communicate via electromagnetic waves [99,100,101] and mechanical/acoustical vibrations [78,102,103,104]. Sound vibrational frequencies, similar to other mechanical waves, can profoundly influence the behavior of different cell types and, ultimately, the functions of different organs [48,49].

Acoustic vibrational emissions ranging from noise to sound, including the sounds of verbal language, can be distinguished based on the degree of intrinsic organization of their wave trains [105,106]. Sound vibrations can be encoded in their specific geometrical shape, and the trains of the sound waves characteristic of each acoustic vibration can directly modulate the morphology of the cytoskeleton [75,76] via mechanical stress or sending relaxation signals up to the nucleus, which normally control movements as the oscillators of proteins present in the cytoplasm [78], thereby positively or negatively influencing mitochondrial trafficking and bioenergetics [76].

The mitochondrial function of mammalian cells can be modulated by acoustic vibration, given the competence of the mitochondria to act as energy portals able to receive, process, and integrate various energetic stimuli and information fields [69,70,71].

Recent studies have investigated the mechanisms involved in the interaction of music at the cellular/subcellular level without the interference of complex human perceptions or personal musical tastes, providing experimental evidence in favor of the hypothesis that music can resonate directly with mammalian cells, modulating their mitochondrial bioenergetic function differently [72,73,74].

A new biofield-based view suggests the existence of a subtle information processing system that is closely involved in the regulation of basic biological processes, from the molecular level to the macro-dimension of the entire organism.

Mitochondria could participate in the “symphony of cellular oscillatory patterns”, acting as dynamic bio-informational “trackers”, transducing and promoting molecular and informational transfer in and out of the organelles and across the cell at the nuclear level, enabling the regulation of the expression of transcriptional regulators and transcription factors [95]. The regulation of cellular energy metabolism was found to occur through circadian subcellular transcriptional oscillations, driving the ATP-dependent energy production by mitochondria [107].

A new framework is emerging that has moved from a purely biochemical point of view, based exclusively on the physical concepts of energy transfer, towards a holistic vision based on the biofield generated by subtle information energies. Science is beginning to decode the principles underlying a more subtle information biology in which specific signaling behaviors could have therapeutic potential.

This new vision emerging in various multidisciplinary fields involving physics, biology, genetics/epigenetics, neuroscience, and psychology should guide scientific research, which so far has provided a fragmented picture of living systems—often with poor connections even between closely related scientific fields—to develop new paradigms and identify unifying principles that lead towards a deeper understanding of the energetic codes of the vibrational flows modulating and interconnecting the physical universe and the living world.

## Data Availability

Not applicable.
